# Plasticity of intestinal gene expression profile signatures reflected by nutritional interventions in piglets

**DOI:** 10.1186/s12864-019-5748-4

**Published:** 2019-05-23

**Authors:** Dirkjan Schokker, Ina Hulsegge, Henri Woelders, Johanna M. J. Rebel

**Affiliations:** 1 Wageningen University & Research Animal Breeding and Genomics, P.O. Box 338, 6700 AH Wageningen, The Netherlands; 2 Wageningen University & Research Animal Health and Welfare, P.O. Box 338, 6700 AH Wageningen, The Netherlands

**Keywords:** Development, Gene expression, Pig, Gut, Plasticity

## Abstract

**Background:**

Immediately after birth, the porcine intestine rapidly develops morphologically, functionally, and immunologically. The jejunum, the second part of the small intestine, is of importance for nutrient uptake and immune surveillance. To study the early postnatal development of the jejunum, a meta-analysis was performed on different transcriptomic datasets. These datasets were acquired from different experimental in-house studies or from experiments described in literature of porcine jejunum mucosa. Gene expression was measured under different experimental interventions, such as nutritional intervention, at various time-points (age).

**Results:**

The studies included in the meta-analysis provided gene expression data for various time-points (piglet ages) for piglets that had received a treatment versus control piglets. In separate studies, treatments were administered to the sow (i.e. amoxicillin), or nutritional supplementation directly to the piglets with medium chain fatty acids (MCFAs), and oral administration of fructooligosaccharides (FOS) or a high dose of zinc-oxide, respectively. In the meta-analysis, genes were grouped into 16 clusters according to their temporal gene expression profiles for control piglets, i.e. the changes of gene expression level over time. Functional analysis showed that these temporal profile clusters had different dominant processes, such as immune related processes or barrier function. Transcriptomics data of treatment piglets was subsequently superimposed over the control temporal profiles. In this way we could investigate which temporal profile clusters (and which biological processes) were modulated by the treatments. Interestingly, not all 16 temporal profiles were modulated.

**Conclusions:**

We showed that it is possible to re-use (publicly available) transcriptomics data and produce temporal gene expression profiles for control piglets with overexpression of genes representing specific biological processes. Subsequently, by superimposing gene expression data from (nutritional) intervention studies we observed deviations from some of these reference profile(s) and thus the plasticity of the system. By employing this meta-analysis approach we highlighted the importance of birth and weaning and the underlying biological processes.

**Electronic supplementary material:**

The online version of this article (10.1186/s12864-019-5748-4) contains supplementary material, which is available to authorized users.

## Background

In piglets, in the first six weeks after birth, a rapid development of the intestine takes place, in which the intestine undergoes morphological, functional, and immunological changes [1, 2]. The ingestion of feed by the neonate induces a series of morphological changes, including increase of villus height and crypt depth, and an increase of the entire gut mucosal surface and its absorptive capacity [3]. The latter is necessary to assure adequate uptake of the nutrients the piglet needs for its growth and maintenance [4]. Functional changes also occur during early life, including pH change in the different intestinal segments and mucin production [1]. The immunological development starts immediately after birth concomitantly with the gut microbiota colonization. The interaction between host cells and microbes is necessary for a proper (intestinal) immune development [5], as germ-free gnotobionts develop a deprived immune system, and lack a proper response against pathogens [6, 7]. Taken together, after birth the gastro-intestinal tract undergoes many changes, and this period is of importance for immune system programming and immune competence in later life. The window-of-opportunity regarding the development of immune competence is; 1) around birth and subsequent first weeks of life, and 2) around the process of weaning. These two life events have profound effect on shaping the gut microbiota and the host’s immune system.

In this meta-analysis we combined results of different studies to investigate the longitudinal gene expression changes in time in jejunum tissue of piglets. The included studies consisted of piglets with a different genetic background, and/or housed in different animal facilities, and being fed different feeds, and thus likely will have a different gut microbiome composition. However, using the normalization tools applied in this meta-analysis these gene expression datasets of jejunum were effectively combined to generate ‘reference’ temporal profiles of gene expressions of control piglets. These reference profiles occur independently of the microbiome differences for “control/regular” housing conditions. Pathway analysis of the genes in each temporal profile cluster was used to further elucidate whether these temporal profiles reflect biological changes in the gut system. Lastly, gene expression data from five specific (nutritional) intervention studies, including Zinc oxide (two studies), medium chain fatty acids, amoxicillin (maternally administered), and fructooligosaccharides (administered neonatal) [8], were superimposed over these reference temporal profiles, to investigate if and how these interventions affect the plasticity of the system, i.e. deviations of the temporal profile clusters and underlying pathways.

## Results

### Overview of the data and associated characteristics

**Fig. 1:**
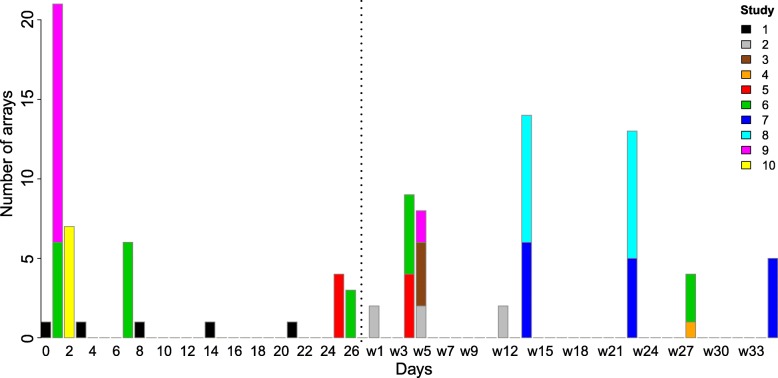
Number of piglets per time-point for the control samples of all studies. The x-axis depicts the time in days, where the vertical dotted line denotes weaning (w). The y-axis depicts the number of arrays. Different colours represent different studies

**Fig. 2:**
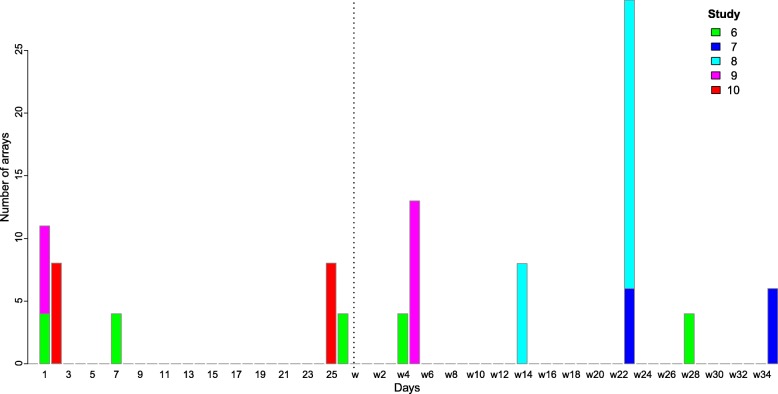
Number of piglets per time-point for the five intervention studies. The x-axis depicts the time in days, where the vertical dotted line denotes weaning (w). The y-axis depicts the number of arrays. Different colours represent different studies, i.e. studies 6 to 10

Jejunum mucosal gene expression datasets from ten different studies were collected to perform a meta-analysis. Animal studies investigating the (gut) development often feature only a limited number of time-points, e.g. two or three, which was also the case for the studies used in this meta-analysis. Combining all studies, data of 103 individual piglets was examined, representing gene expression levels at 18 different time-points ranging from day of birth (d0) until 35 days post weaning (w35) (Fig. 1). The time points were adequately distributed, with the longest gap between time-points being 9 days long. The number of piglets per time-point ranged from 1 to 21 piglets from 1, 2, or 3 different studies per time point, but many time-points have at least 5 piglets. In four of the used studies (all performed in our institute) control piglets were compared with piglets that received a treatment directly, or indirectly through the mother. Together, these five studies provided gene expression data for eleven time-points: d1, d2, d7, d25, d26, w4, w5, w14, w23, w28, and w35 (Fig. 2), with data from at least 4 piglets per time point.

### Clustering

The 7189 genes were grouped into 16 clusters on the basis of their temporal expression profiles. The determination of the appropriate cluster number was based on the minimum centroid distance (Additional file 1: Figure S1), where this minimum centroid distance can be used as a cluster validity index. These profiles can differ from each other in various ways. For example, on the basis of specific time-points that are higher or lower than the rest of the profile, or on the basis of an overall positive or negative trend (increase / decrease in time), or the same but then pre-weaning or post-weaning.

**Fig. 3:**
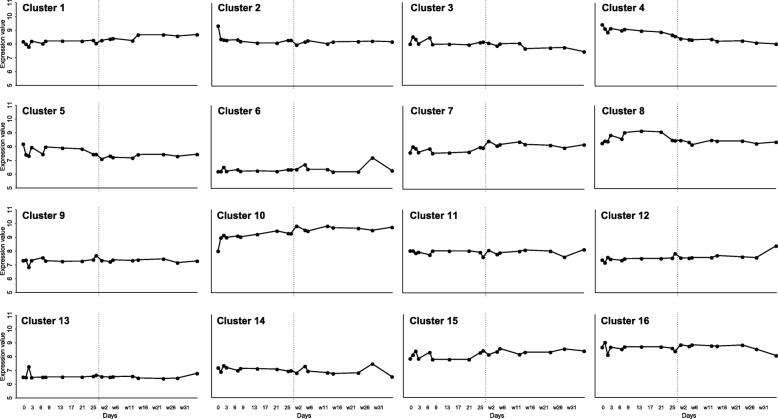
Temporal gene expression profiles (clusters) based on the control studies. In each graph the x-axis depicts time in days, where ‘w’ stand for weaning and is illustrated by a vertical dotted line. The time period runs from day 0 (birth) to 35 days post–weaning. The y-axis denotes the (normalized) expression value, ranging from 5 to 11. The scale of both x- and y-axis are similar for each graph. Each graph represents one of the sixteen clusters, which are based on Self-Organizing Maps method

**Fig. 4:**
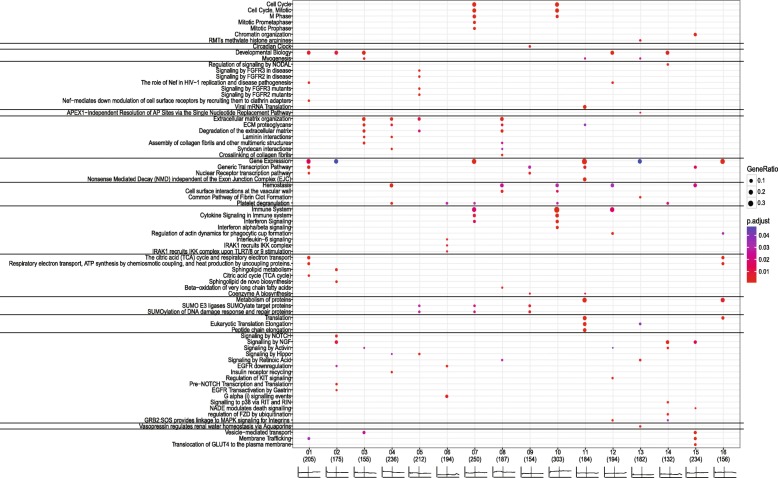
Pathway enrichment results of all gene cluster. The dot size is proportional to the percentage of genes in the cluster belonging to that particular pathway. Coloured dots correspond to the p-value (< 0.1), where blue corresponds to 0.05 and red to below 0.01

The number of genes allocated to each cluster varied from 337 (cluster 14) to 561 (cluster 5), Table 1 depicts an overview of all 16 clusters. The temporal gene expression profiles of individual genes of all 16 clusters is shown in Additional file 2: Figure S2. However, we focused on the average expression profile of each cluster (Fig. 3). Subsequently, to identify which processes were linked to these clusters a pathway enrichment was performed for all cluster, this resulted in a total of 1067 significant pathways, ranging from 30 to 194 pathways in a single cluster (Table 1). Significant pathways for each clusters are highlighted in Fig. 4, this is based on the *p*-value and gene ratio of each pathway within a cluster.

**Table 1:** Total number of genes in each cluster

Cluster	#genes	#annotated genes^a^	% annotated	# pathways	# highlighted pathways
1	450	205	45.6	59	10
2	439	175	39.9	72	9
3	403	155	38.5	44	9
4	560	236	42.1	45	8
5	561	212	37.8	54	9
6	468	194	41.5	48	6
7	539	250	46.4	115	12
8	375	187	49.9	65	10
9	392	154	39.3	30	6
10	531	303	57.1	194	10
11	389	184	47.3	96	11
12	424	194	45.8	85	8
13	432	182	42.1	31	8
14	337	132	39.2	45	8
15	537	234	43.6	45	8
16	352	156	44.3	39	6

^a^These gene names were used as input in ReactomePA

### Superimposing gene expression data from intervention studies

**Fig. 5:**
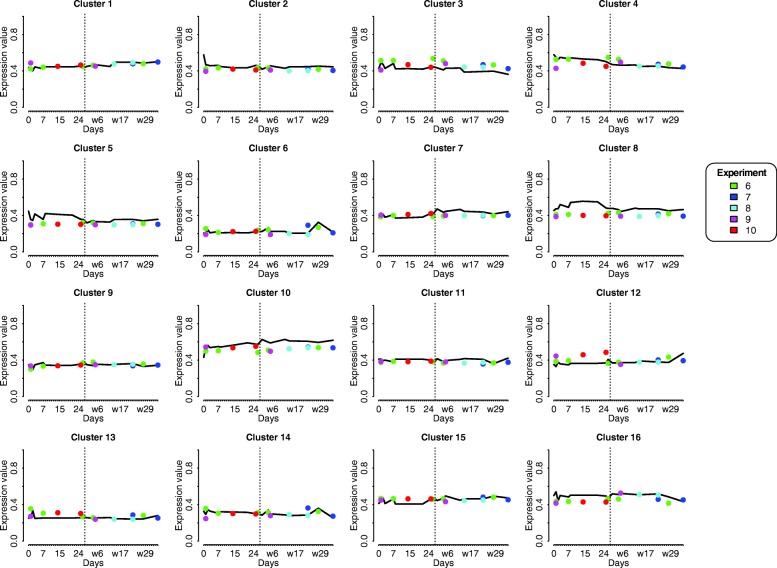
Intervention gene expression data of four experiments superimposed on references time-profiles. All 16 clusters showing the reference profiles are depicted. In each graph the x-axis depicts time in days, where ‘w’ stand for weaning. The y-axis denotes the (normalized and scaled) expression value. The five experiments were 6 (green), 7 (blue), 8 (cyan), 9 (magenta), and 10 (red)

Per cluster and per (nutritional) intervention study, the mean expression of the genes in the cluster was superimposed over the reference temporal profile of that cluster, indicating only small deviations of mean gene expression per cluster from the respective reference profile (Fig. 5). For a number of clusters, the deviations appeared to be consistently up or down for the various time points, and, strikingly, also for the various intervention studies, indicating that these treatments resulted in similar up- or down-regulation of processes underlying these clusters. In cluster 3, the mean gene expression (mean over all genes of cluster 3 over all piglets per intervention) for the four different interventions was consistently higher than the reference profile. In contrast, lower gene expression in the four different interventions is shown in clusters 2, 5, 7, 8, 10, 11, and 16. Clusters 4, 9, and 13, show no deviation of gene expression in response to the different interventions.

## Discussion

In this study we have combined ten datasets with gene expression data of intestinal mucosal tissue at various time points to identify a reference gene data set of different temporal profiles of gene expression in young pigs. The jejunum intestinal tissue was used, because it plays an important role in the absorption of nutrients and minerals [4], has immunological activity [9], and is involved in the immune system programming in early life as well as metabolic programming [10, 11]. Also for the jejunum enough datasets for a this meta-analyses, were available and therefore a more meaningful analysis could be performed in comparison to ileum. With this approach, we distinguished 16 clusters of genes with similar temporal expression profiles. These clusters were subjected to functional analysis to identify the underlying biological processes. This resulted in 16 number of clusters with clear associated function(s), including cell cycle, immunological, or extracellular matrix. Subsequently, we investigated the impact of nutritional interventions on the plasticity of the gut system, i.e. the deviation from the established reference profile in time.

### Current limitations of this Meta-analysis

Meta-analyses of gene expression studies often focus on static data, comparing two states, e.g. control versus diseased animals, on a single time-point. Combining the available data-points on a single time-point of such studies in a meta-analysis approach increases the power and allows one to draw (more) unbiased conclusions about specific genes involved in a particular disease [12], but does not capture development or (immune) reactions over time. To our knowledge, the present study is the first meta-analysis aimed at providing temporal expression profiles of intestinal mucosa development in piglets and subsequently investigated the impact of different nutritional interventions on the plasticity of the gut system. By combining different existing tools and scripts, we generated a customized-analysis-pipeline. In the current study, data on time-points beyond 100 days of age, available in a few studies, were not included, as coverage of that upper range of piglet ages was considered insufficient, i.e. the gaps between the last included time-point (‘w + 35’) and the next time-point was up to 21 days, while the gaps between the time-points included in this meta-analysis was maximally seven days. However, in the selected time period of the current study, 0 to 63 days, in 4 out of the 18 time-points a single array of a single study (days 0, 3, 8, and 14) represents that time-point. Because of this low coverage of time-points, gene expression levels of specific time-points may have been skewed by an individual experiment. Unfortunately, more datasets were not available at the time of analysis. By linking the underlying datasets with the reference profiles at each time-point, we have gained some insight in the skewing by dataset. We have identified several specific cases in which deviations in the reference profile could be assigned to an individual dataset, e.g. day 1 is solely relying on a single piglet from one specific study.

### Time-points with impact on intestinal development

The temporal patterns showed fluctuations in the different clusters around two periods, i.e. day of birth and day of weaning, also defined as a putative window-of-opportunity where intestinal development is defined. Early life nutrition and/or management have a large impact on the gut system, around birth as well as weaning [13, 14]. During these two transition periods, birth and weaning, the gut system encounters new antigens. At birth the gut system encounters mother’s (sows) milk and microbiota will colonize the entire gastrointestinal tract. Whereas at weaning solid feed is introduced and animals will encounter more stressors, due to the new living environment with other piglets that were not littermates, as well as placement without their mother (sow). When analysing the functions of genes residing in clusters showing the largest fluctuations at birth or weaning, processes like gut immune system development and morphological development were observed. These processes that change around birth and around weaning were especially observed in clusters 7 and 10 for immune related processes and in clusters 2, 3, 4, 5, and 8 for morphological development. From these clusters it is concluded that day of birth and day of weaning are of importance for the development of immune and morphological processes. In the other clusters a more stable flat pattern, not dependent on age was observed, in these clusters “maintenance” processes were observed.

### Immune system development in the gut

Immune system programming develops in two specific periods as shown in the different clusters, although the ontogeny of immune cells starts in the foetal period [15], further programming of the gut associated immune system occurs after birth and is especially observed in cluster 7 and 10, and to a lesser extent in cluster 6. This programming after birth occurs because piglets encounter feed (sow’s milk) and environmental challenges for the first time [16] and simultaneously microbial colonization occurs [17]. The gut immune system develops by interaction with the resident microbiota and the environment. This programming process take place immediately after birth, nevertheless the interaction between the host and microbiota will continue life-long. At weaning the piglets encounter different levels of stress due to a new environment, without their mother, and after weaning piglets eat solely solid feed. Especially, the latter will give a second boost to the gut immune system development, because novel antigens are introduced together with a change in microbiota composition which are both of importance for the intestinal immune development and response as tolerance and non-tolerance is further developed. Here, we observed the association of temporal gene expression profiles to immune system processes. The main associated clusters were 6, 7, and 10. Where cluster 10 had an overall high expression level pre- and post-weaning and the temporal profile showed an initial sharp increase directly after birth, followed by a steady rise in expression level over time. Whereas, cluster 7 follows an increase of expression in time, and from birth to weaning a small increase is observed, after weaning a small sharp increase in expression is observed followed by a plateau. Cluster 6 showed an overall flat expression profile in time, with increases in expression around day 2, and 5 and 28 days after weaning. The overall gene expression profiles of clusters 7 and 10, specifically the high expression immediately after birth, has also been observed in studies with calves [18] and chicken [19], moreover similar evidence is observed in pig studies [20–22]. In chicken, it has been shown that genes involved in intestinal immune development are increasing immediately post-hatch [19]. In addition, this high base level of immune related processes is in line with the fact that the gut is the largest immune system and interacts with the environment. [23]. In cluster 7, immune system processes are dominant, as well as cell cycle related processes. Taken together, these two clusters show an increase in gene expression as function of time, in a timeframe which takes place from birth to the post-weaning stage [24, 25]. Which was not observed in the chicken and calves studies, probably because no weaning occurred in chicken and in calve study.

### Morphological development of the gut

The morphology of the intestine and its development in early life are important for the uptake of nutrients as well as the barrier function. For example affected intestinal tissue cause a decreased nutrient absorption, as well as a greater risk of translocation of pathogens possibly leading to infection. As stated earlier, we also expected fluctuations in gene expression around birth and weaning, as these phenomena have a great impact on the gut morphology and in turn on the maintenance. Multiple clusters, 2, 3, 4, 5, 8, 12, and 14, showed a high basal expression level before weaning and low basal levels of expression after weaning, genes in these clusters were dominantly associated to developmental and morphological processes. This is in line with the immediate morphological development of the gut after birth, where suckling boosts intestinal growth and modifies brush border digestive functions [3] and the overall development of the piglet, simultaneously colonization with bacteria occurs influencing proliferation and apoptosis of the resident enterocytes [26]. This includes rapid proliferation of the intestinal tissue, meaning that the cellular structure, i.e. extracellular matrix [27, 28], will be modified to support this morphological change. It was expected that the gene expression of these morphological processes would decrease in time, here we have verification of this gut maturation process reflected by the (average) gene expression.

### Maintenance of the gut

Clusters with a relatively flat pattern in time and generally only showed small fluctuations could often be linked to maintenance processes of the gut. This encompasses clusters 1, 9, 11, 13, 15, and 16, where we observed biological processes such as metabolism of proteins, gene expression, the citric acid (TCA) cycle, and hemostasis were age and transitions periods of the piglets did not influence these processes. These processes are of course a necessity and often showed a lower average gene expression level compared to the ‘immune’ and ‘morphological’ clusters. This could be due to the fact that more data was available in the more crucial periods where growth and development are key for the performance of the piglets, and therefore relatively lower expression is observed for these ‘maintenance’ processes.

### Effect of interventions in immune and morphological related processes

To investigate to what extent these identified reference gene expression profiles could be modulated, we superimposed several in-house datasets in which (nutritional) interventions were administrated to the piglets, directly or indirectly (via sow). A striking result was that the gene expression from ‘treatment piglets’ in the separate intervention studies, when superimposed on the reference temporal profiles of the 16 clusters, showed similar (up or down) deviations from these reference profiles. This was observed in clusters 2, 4, 5, 7, 8, 10, 11 and 16, which showed down-regulation before and/or after weaning. Whereas, clusters 3, 12, and 13 showed up-regulation before and/or after weaning. Lastly, clusters 1, 6, and 15 showed almost no change from the reference profile, these latter 3 clusters were the more maintenance related stable gene expression clusters. While the other maintenance clusters 9, 11, 13 and 16 were influenced by interventions and not by age. Thus the age-influenced immunological (7, 10) and morphological clusters (2, 3, 4, 5, 8) were also influenced by the intervention and show the plasticity of the gut system.

Interestingly, all intervention data-points were below the reference profile in cluster 10, which was enriched in processes related to immune system programming. Thus the four interventions (five studies); amoxicillin, MCFA, Zinc oxide, and FOS, all showed down-regulation of immune related processes, for amoxicillin, MCFA, and zinc oxide it is known that these have anti-bacterial properties [29–33]. In turn, this decrease in gene expression of immune related genes could influence the gut immune system programming [16, 34] and consequently the gut (microbiome) homeostasis. For FOS, in mice studies have shown that it regulates immunoglobulin A and polymeric immunoglobulin receptor expression in the small intestine [2]. In mice, it was also shown that alterations of the microbiome in early life affected the metabolic status in later life [35]. It may be speculated that this could also affect immune functioning later in life. Thus, we may hypothesize that the decreased gene expression of immune-related genes induced by the anti-bacterial treatments may have consequences for immune competence of the piglets in their later life, although part of the immune processes cluster 7 were hardly affected, meaning that these processes are more robust and less affected by these (nutritional) interventions.

Another observation was that higher gene expression values in the intervention groups, compared with the reference profile, were only observed for cluster 3, which is enriched in processes related to development or to extracellular matrix (ECM). This suggests that the (nutritional) interventions induce the genes in this cluster compared to the reference gene expression profiles in the small intestine. However, for cluster 4 and 8 that also have many genes involved in ECM processes, show down-regulation compared to the reference profile.

Gene expression of three clusters (4, 9, and 13) were not modulated due to interventions. In cluster 4 the dominant process of the genes was ECM and for cluster 13 and 9 more generic maintenance gene expression processes were observed. Whereas, genes from cluster 9 were involved in Small Ubiquitin-like Modifier (SUMO) processes. SUMOylation, i.e. modifications of proteins by a SUMO, are important in several functions, including protein stability, transport from the nucleus to the cytosol, and transcriptional regulation. Possibly, expression of these processes in these three clusters are indispensable for survival of the cell/tissue, and therefore less variable.

This meta-analysis has shown that it is possible to re-use transcriptomic data and investigate temporal profiles in light of important biological processes in the gut. Furthermore, by superimposing transcriptomic data from (nutritional) intervention experiments we have shown that certain processes could be modulated, whereas others could not.

## Conclusion(s)

Meta-analysis of intestinal gene expression data was technically successful and allowed to distinguish different clusters of genes with different temporal expression profiles. Subsequent functional analysis of these clusters revealed functional processes coupled to specific temporal profiles on specific ages of the pigs, time of birth and weaning. Some processes were not dependent on age, and were stable over the whole period.

After the (nutritional) intervention, accompanying gene expression profiles were consistently lower, similar, or higher than the respective reference gene expression profile. Only three clusters did not change due to an intervention, meaning that most of the intestinal developmental processes could be changed by different intervention, but some of the processes in the development of the intestine are stable as shown in this meta-analysis.

Taken together, this study could help in formulating new hypotheses regarding the gut development and its plasticity. For example which biological processes could be modulated by a nutritional intervention to have an increased performance.

## Methods

### Datasets acquisition

Two major public microarray repositories, Gene Expression Omnibus (GEO, National Center for Biotechnology Information [36, 37]) and *ArrayExpress* (AE, European Bioinformatics Institute [38, 39]) were queried for experiments with species *Sus scrofa* (pig), and the keyword ‘jejunum’. The R package *GEOmetadb* (v1.28.0) [40] was used for searching GEO and the package *ArrayExpress* (v1.28.1) [41] for searching ArrayExpress. The raw data of the control samples (samples of piglets that have not undergone any treatment) of experiments acquired by the search query were retrieved from the repositories (Table 2). Furthermore, datasets of four experiments from our group (Wageningen UR, Feed4Foodure: “Voeding, Darmgezondheid en Immuniteit” research programme) were used as well (Table 2). In addition to the above mentioned control datasets, from our in-house studies 6–10 we also have gene expression data on specific nutritional interventions (Table 3). Study 6 investigated the effect of a maternal antibiotic intervention (administered one week before expected day of farrowing until farrowing) on the gut development and microbiota in the offspring, study 7 investigated the effects of a high level of dietary zinc two weeks after weaning on intestinal microbiota and mucosal gene expression in piglets, whereas study 8 investigated a high level of dietary zinc 1 in the first and/or second week after weaning, and study 9 investigated the effect of neonatal (directly to piglets) and maternal (via sow) administration (one week before expected day of farrowing until farrowing) of medium-chain fatty acids (MCFAs) on intestinal microbiota and mucosal gene expression in piglets. Study 10 investigated the effect of administrating fructooligosaccharides from day 2–14 after birth on intestinal microbiota and mucosal gene expression in piglets.

**Table 2:** Overview of the studies included in the meta-analysis

Study	Accession number	Days	platform	Year	Publication
1	GSE13456	0 (directly after birth), 3, 8, 14, 21	Affymetrix Porcine Genome Array	2008	[40]
2	GSE13457	24, 28, 35	Affymetrix Porcine Genome Array	2008	–
3	GSE22596	~ 4 weeks old (~ 28 days)	Affymetrix Porcine Genome Array	2010	[41]
4	E-MEXP-2198	± 56	Affymetrix Porcine Genome Array	2009	[42]
5	GSE48050	25^a^	Agilent-026440 *Sus scrofa* (Pig) Oligo Microarray v2 (Probe Name version)	2013	[43]
6	VDI-2	1, 7, 26, 30, 54	Agilent-035953 Sus scrofa Array	2013	In-house
7	VDI-5.1	42, 51, 63	Agilent-035953 Sus scrofa Array	2014	In-house
8	VDI-5.2	42, 51	Agilent-035953 Sus scrofa Array	2015	In-house
9	VDI-12	1, 31	Agilent-035953 Sus scrofa Array	2016	In-house
10	VDI-3	2	Agilent-035953 Sus scrofa Array	2014	[8]

^a^8 piglets on day 25, 4 of which had been weaned on day 21 and 4 had not been weaned before being sampled

**Table 3:** Datasets with a (feed) intervention

Study	Accession number	Days	Feed intervention	Platform	Year
6	VDI-2	1,7,26,30,54	Maternal administration of amoxicillin	Agilent-035953 Sus scrofa Array	2013
7	VDI-5.1	51,63	Zinc oxide (d14 to d23 post weaning)	Agilent-035953 Sus scrofa Array	2014
8	VDI-5.2	42,51	Zinc oxide (d0 to d14 and/or d14-d23 post weaning)	Agilent-035953 Sus scrofa Array	2015
9	VDI-12	1,31	Maternal administration of medium-chain fatty acids	Agilent-035953 Sus scrofa Array	2016
10	VDI-3	14,25	Administration fructooligosaccharides (d2–14)	Agilent-035953 Sus scrofa Array	2014

#### Array quality check

Quality assessment for the raw data was carried out by using the R package *arrayQualityMetrics* (v3.18.0) [42]. Arrays with three potential problems, i.e. three stars in the summary table from the *arrayQualityMetrics* report, were excluded from further analyses. For the Affymetrix Porcine Genome Array arrays the ‘.CEL’ file were imported with the *cdf* package based on the Affymetrix mappings in the R package *affy* (v1.46.1). Each Affymetrix Porcine Genome Array dataset was background adjusted, normalized, and log2 probe-set intensities were calculated using the Robust Multichip Averaging (RMA) algorithm in *affy* package [43, 44]. The Agilent-026440 *Sus scrofa* (Pig) Oligo Microarray v2 (probe name version) and Agilent-035953 *Sus scrofa* Array arrays were background corrected (method = “normexp” and offset = 1) using functions from the R package *Limma* (v3.18.13) [45, 46]. Subsequently, quantile normalization of the data was performed between the arrays. Duplicate probes were averaged by using the *avereps* function.

#### Annotation

The Affymetrix Porcine Genome Array annotation file, Affymetrix Porcine Annotation, Revision 6 was used [47]. For Agilent array data, an in-house manually curated revised version of Agilent-035953 *Sus scrofa* Array (GPL18045; http://www.ncbi.nlm.nih.gov/geo/query/acc.cgi?acc=GPL18045) was used (May 2014). The porcine gene symbols were converted to human gene symbols using BioMart Gene ID Converter (http://www.biomart.org/) [48], this conversion is performed because the annotation of the human genome is more advanced compared to the pig genome. The human gene symbols were checked on the following website; http://www.genenames.org/cgi-bin/symbol_checker (8-3-2016).

### Data integration

**Fig. 6:**
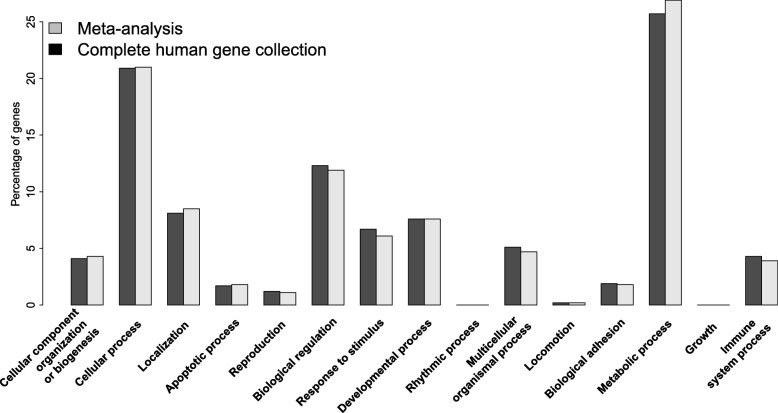
Functional annotation of the genes included in the meta-analysis compared to the complete human gene collection. The x-axis depicts different functional categories, whereas the y-axis depicts the percentage of genes included. Dark-grey represent the complete human gene collection, whereas the light-grey represent the current meta-analysis

The first step of the dataset integration was to extract a set of gene names/symbols, based upon the probe sequences which were common across the three different platforms, Affymetrix, Agilent-026440, and Agilent-035953. In total, 7189 (annotated) genes were common across the three platforms and these genes were used for further analyses, while those absent in one of the platforms were excluded from further analysis. By combining different experiments with different platforms, a certain amount of gene expression information will be lost, i.e. the non-common genes. To identify possible loss of functional annotations due to loss of genes included for the analysis, we performed a large-scale gene function analysis (Panther, v10.0; release date April 25, 2015) [49]). We compared the common genes across platforms with all the human genes in Gene Ontology (GO) within the category ‘Biological Process’. The 7189 common genes across platforms had a proper representation of all biological functions (Fig. 6). The second step was that the expression values of the common genes were transferred to a unified scale by using the *normalizeBetweenArrays* and *removeBatchEffect* functions from the R package *Limma* [46].

### Clustering and time course profile

In the studies included for the meta-analysis the weaning day varied from d21 to d28. To compare the post-weaning gene expression data from these studies, we standardized the time axis after weaning by using day post weaning (denoted by the use of ‘w’ before the age in days). Expression values for the time series were soft clustered using a fuzzy c-means algorithm via the R package *Mfuzz* (v2.30.0), which is suggested for microarray time-course data [50]. Since the clustering was performed in Euclidian space (encompassing the *x*, *y*, and *z* planes), the expression values of genes were standardized using the standardize function of R, so that the mean expression for each gene is zero with a standard deviation of one. This ensures that vectors of genes with similar changes in expression are close in Euclidean space. An optimal cluster number (c) of 16 was determined by visual inspection for a plateau in the minimum centroid distance using the *Dmin* function (Additional file 1: Figure S1) [51]. The optimal fuzzifier (m) of 1.130857 was calculated using the *mestimate* function. A membership value between 0 and 1 to each gene which gives an indication as to how closely that gene matches the cluster core.

Statistical analysis and visualization of functional profiles for the different gene clusters were carried out with the R package *clusterProfiler* (v1.9) [52]. First, the human gene symbols were converted to Entrez gene identifiers using the function *bitr* by using the Bioconductor annotation package *org. Hs.eg.db* (v3.2.3). Subsequently, enriched functional categories of each gene cluster was calculated by using the *compareCluster* function. Within the *compareCluster* function we employed the *enrichPathway* function from the package *ReactomePA* (v1.14.4) [53] to compare the biological themes from the Reactome pathway perspective [54]. Here, we used the complete (human) gene set as background.

## Additional files

Additional file 1: **Figure S1.** Determining an appropriate cluster number using minimum centroid distance. Based on this calculation we have set the number of clusters to 16 (dotted vertical line). (PDF 2 kb)

Additional file 2: **Figure S2.** Clusters of gene expression data based on the control studies. The x-axis depicts the time in days, where the vertical dotted line denotes weaning (w). The y-axis depicts the normalized gene expression value. The black solid line depicts the mean expression profile of a cluster. High membership value is denoted by red and purple lines, whereas low membership value is denoted by yellow or green lines. (PDF 480 kb)

## Abbreviations

ECM: Extracellular matrix
FOS: Fructooligosaccharides
MCFAs: Medium chain fatty acids
