# Supplementary figures and images for: Plasticity of intestinal gene expression profile signatures reflected by nutritional interventions in piglets
Source: BMC Genomics. 2019 May 23;20:414. doi: 10.1186/s12864-019-5748-4 (PMC6533718; doi:10.1186/s12864-019-5748-4)

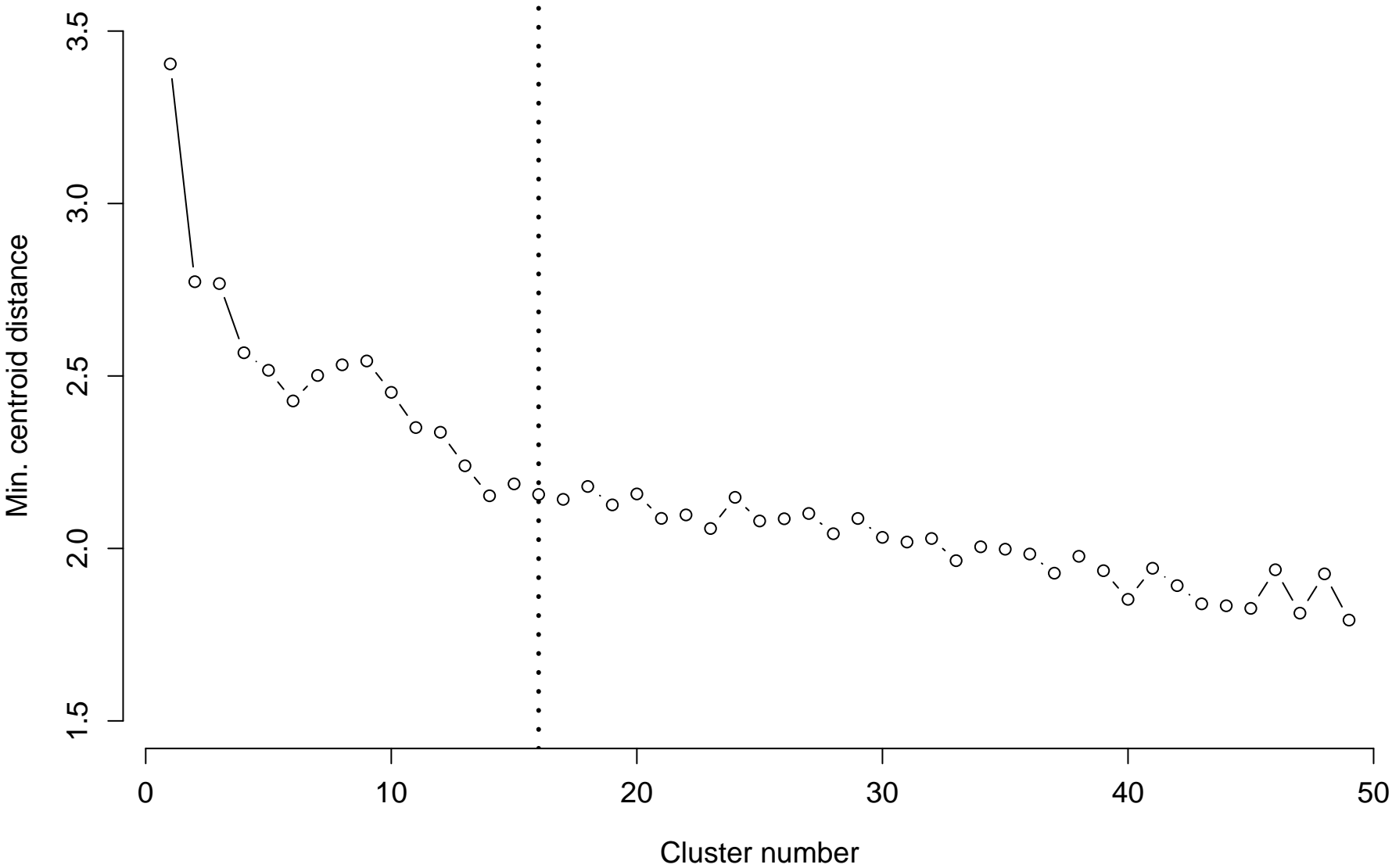

Supplement: Supplementary file 1 — Figure S1. Determining an appropriate cluster number using minimum centroid distance. Based on this calculation we have set the number of clusters to 16 (dotted vertical line). (PDF 2 kb) [file 12864_2019_5748_MOESM1_ESM.pdf]

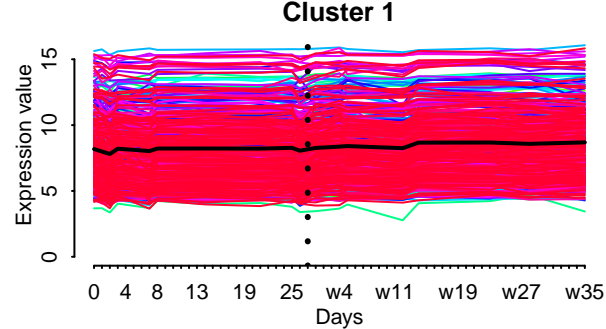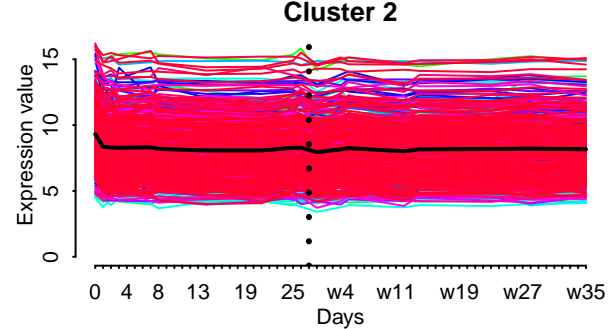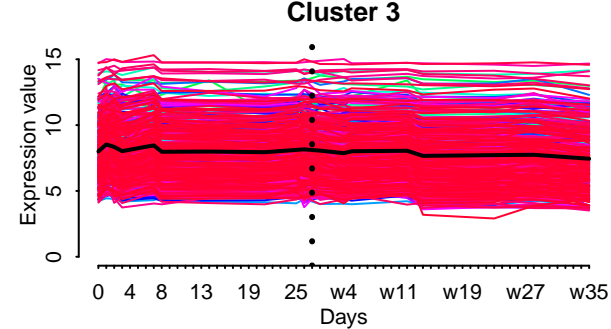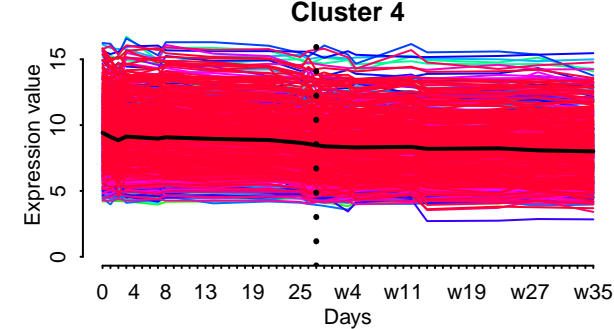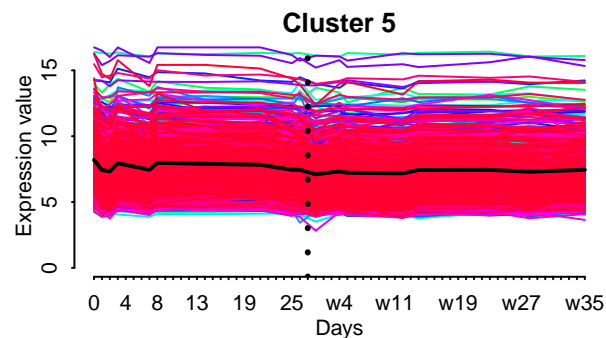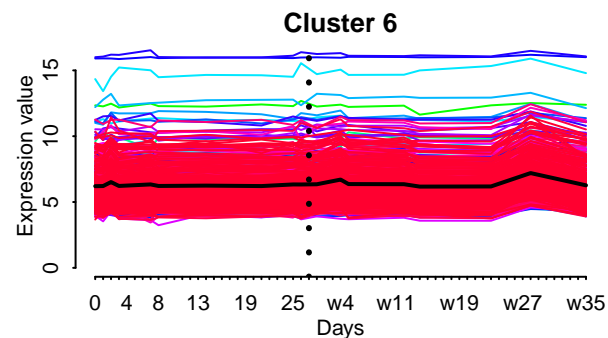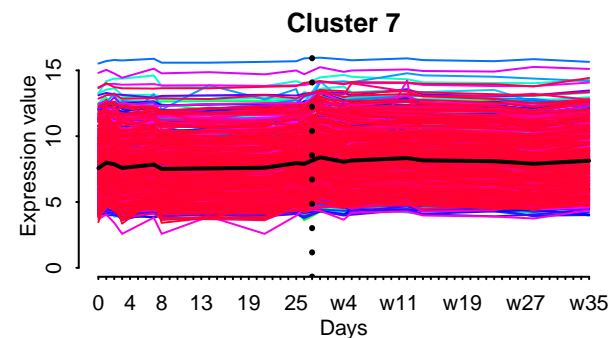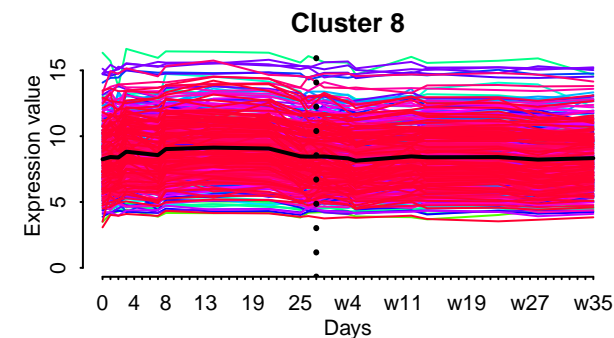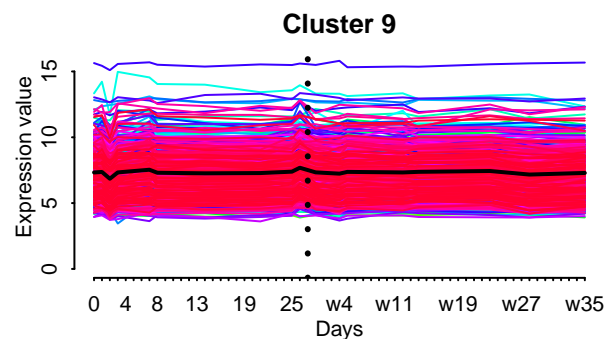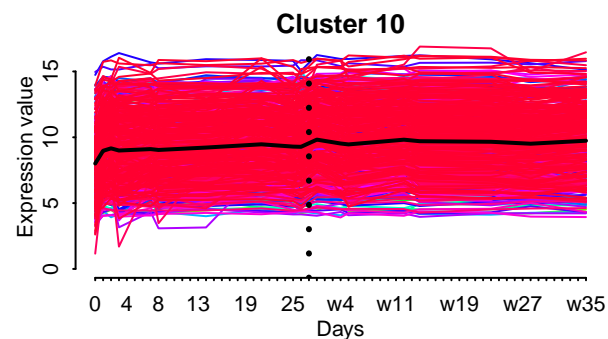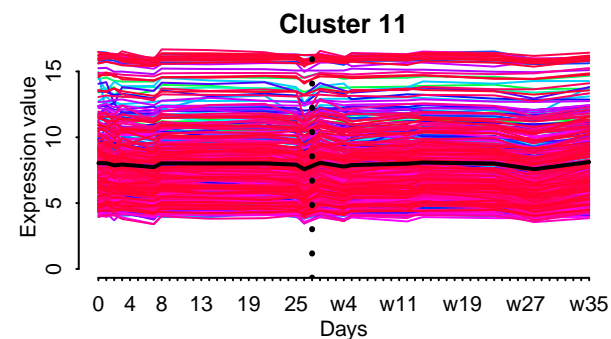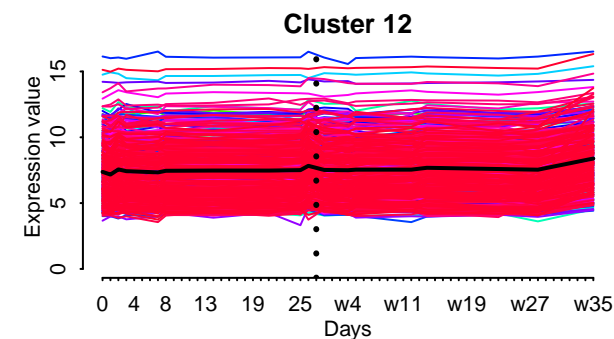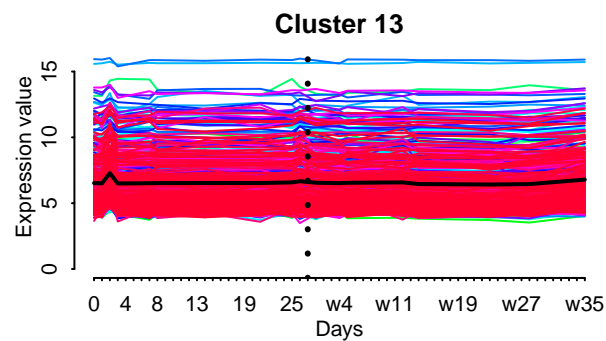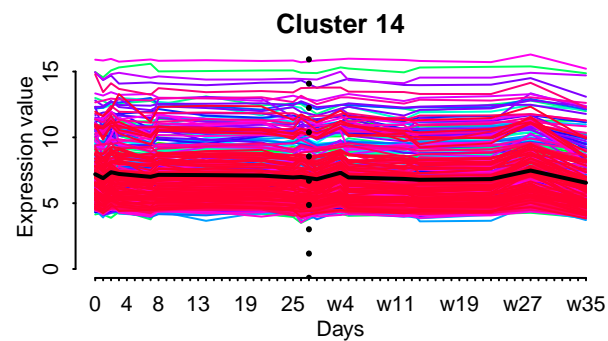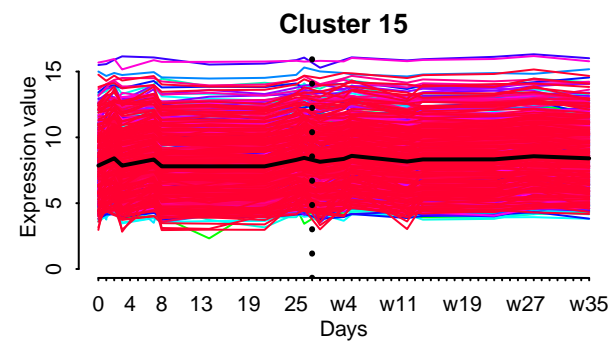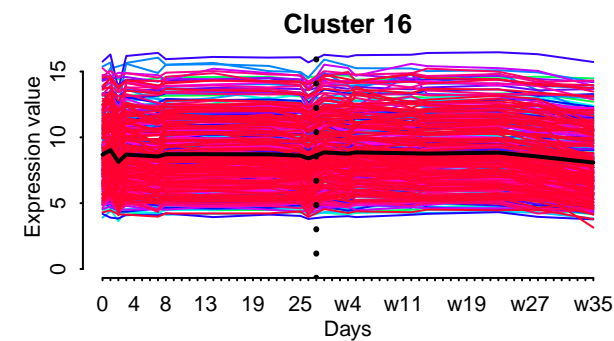

Supplement: Supplementary file 2 — Figure S2. Clusters of gene expression data based on the control studies. The x-axis depicts the time in days, where the vertical dotted line denotes weaning (w). The y-axis depicts the normalized gene expression value. The black solid line depicts the mean expression profile of a cluster. High membership value is denoted by red and purple lines, whereas low membership value is denoted by yellow or green lines. (PDF 480 kb) [file 12864_2019_5748_MOESM2_ESM.pdf]
